# Heterogeneous neurodevelopmental disorders in children with Kawasaki disease: what is new today?

**DOI:** 10.1186/s12887-019-1786-y

**Published:** 2019-11-04

**Authors:** Chien-Heng Lin, Wei-De Lin, I-Ching Chou, Inn-Chi Lee, Syuan-Yu Hong

**Affiliations:** 1 Division of Pediatrics Pulmonology, China Medical Univeristy Children’s Hospital, Taichung, Taiwan; 2 Department of Biomedical Imaging and Radiological Science, College of Medicine, China Medical University, Taichung, Taiwan; 3 Department of Medical Research, China Medical University Hospital, Taichung, Taiwan; 4 Division of Pediatrics Neurology, China Medical Univeristy Children’s Hospital, Taichung, Taiwan; 5 Graduate Institute of Integrated Medicine, College of Chinese Medicine, China Medical University, Taichung, Taiwan; 6 Department of Pediatrics, Chung Shan Medical University Hospital and Institute of Medicine, School of Medicine, Chung Shan Medical University, Taichung, Taiwan

**Keywords:** Kawasaki disease, Neurodevelopmental disorders, Children, Epilepsy, Tourette syndrome

## Abstract

**Background:**

Kawasaki disease (KD) is a common vasculitis of childhood in East Asia. The complications of KD ascribed to long-term cardiovascular sequelae are considerably diverse. Although studies have investigated neurodevelopmental problems following KD in the past few decades, they have reported inconsistent conclusions. This study investigated potential epilepsy and associated neurodevelopmental disorders (NDDs) following KD in Taiwanese children.

**Methods:**

We retrospectively analyzed the data of children aged < 18 years with clinically diagnosed KD from January 1, 2005, to December 31, 2015. These patients were followed up to estimate the prevalence of epilepsy and associated NDDs in comparison with the prevalence in general pediatric population in Taiwan and worldwide.

**Results:**

A total of 612 patients with an average age of 1.6 years were included. The prevalence of associated NDDs was 16.8% (*n* = 103/612) in the study group, which consisted of epilepsy, intellectual disability (ID), autism spectrum disorders, Tourette syndrome (TS), attention deficit hyperactivity disorder, (ADHD), and others. Moreover, children with KD had a higher prevalence of epilepsy and TS in both Taiwan and worldwide (epilepsy: 2.61% in the KD group vs 0.33% in Taiwan and 0.05–0.8% in worldwide, *p* < 0.05; TS: 2.77% in the KD group vs 0.56% in Taiwan and 0.3–1% in worldwide, *p* < 0.05). The prevalence of ID, ADHD, and developmental language disorders was not significantly different between our study patients and those in Taiwan or worldwide.

**Conclusions:**

Results revealed a higher prevalence rate of NDDs, especially epilepsy and TS, in Taiwanese children with KD than in the general pediatric population in Taiwan. However, these NDDs could be heterogeneous. Children diagnosed with KD were followed up because they had a higher risk of heterogeneous NDDs.

## Background

Kawasaki disease (KD), also known as mucocutaneous lymph node syndrome, is a common vasculitis of childhood, particularly in East Asia. The complications of KD, probably ascribed to long-term cardiovascular sequelae, are considerably diverse [1]. However, in addition to cardiac complications [2], noncardiac complications may affect children with KD [3, 4]. In KD, medium-sized muscular arteries, rather than small vessels, are most commonly affected. Hence, complications relevant to organs outside the heart but abundant in such vascular beds have been observed over the past few decades [3], including urinary or renal disease [5], gastrointestinal abnormalities, and those related to the central nervous system [6, 7].

Among complications of KD, few studies have investigated those related to the central nervous system, but they have reported inconsistent conclusions regarding their long-term neurological problems [7–9]. Little is known regarding the correlation between neurodevelopmental disorders (NDDs) and KD and their different prevalence rates.

We conducted this retrospective observational study between January 1, 2005, and December 31, 2015, and followed up until December 31, 2018 to investigate the occurrence of potential epilepsy and associated NDDs following KD in Taiwanese children. The findings of this study can provide extensive insights into KD-related NDDs.

## Methods

### Data sources and study population

In this retrospective cohort study, we analyzed patients aged < 18 years with clinically suspected KD. The following preliminary inclusion criteria were based upon diagnostic criteria for KD between January 1, 2005, and December 31, 2015 [10].

The presence of fever lasting at least 5 days without any other explanation combined with at least four of the five following criteria:

1. Bilateral bulbar conjunctival injection
2. Oral mucous membrane changes, including injected or fissured lips, injected pharynx, or strawberry tongue
3. Peripheral extremity changes, including erythema of the palms or soles, edema of the hands or feet (acute phase), and periungual desquamation (convalescent phase)
4. Polymorphous rash
5. Cervical lymphadenopathy (at least 1 lymph node > 1.5 cm in diameter).

A comprehensive medical record review was strictly enforced to exclude children who had epilepsy, neurologic, metabolic, autoimmune (other than KD), or any other congenital disorders before the onset of KD. Other exclusion criteria were as follows:

1. Loss of contact with a patient during the follow-up period
2. Patients who developed NDDs or epilepsy with documented etiology or followed by a causative event; for example, central nervous system infections, copy number variations, or single gene mutations, which are related to epilepsy and NDDs.
3. Patients who were born relatively preterm (< 32 weeks)
4. Patients who had a perinatal history of hypoxic ischemic encephalopathy or birth asphyxia and congenital infection.
5. Patients who had a history of traumatic brain injury.
6. Maternal medication use during pregnancy; for example, heavy smoking, drinking, and drug abuse.

**Fig. 1:**
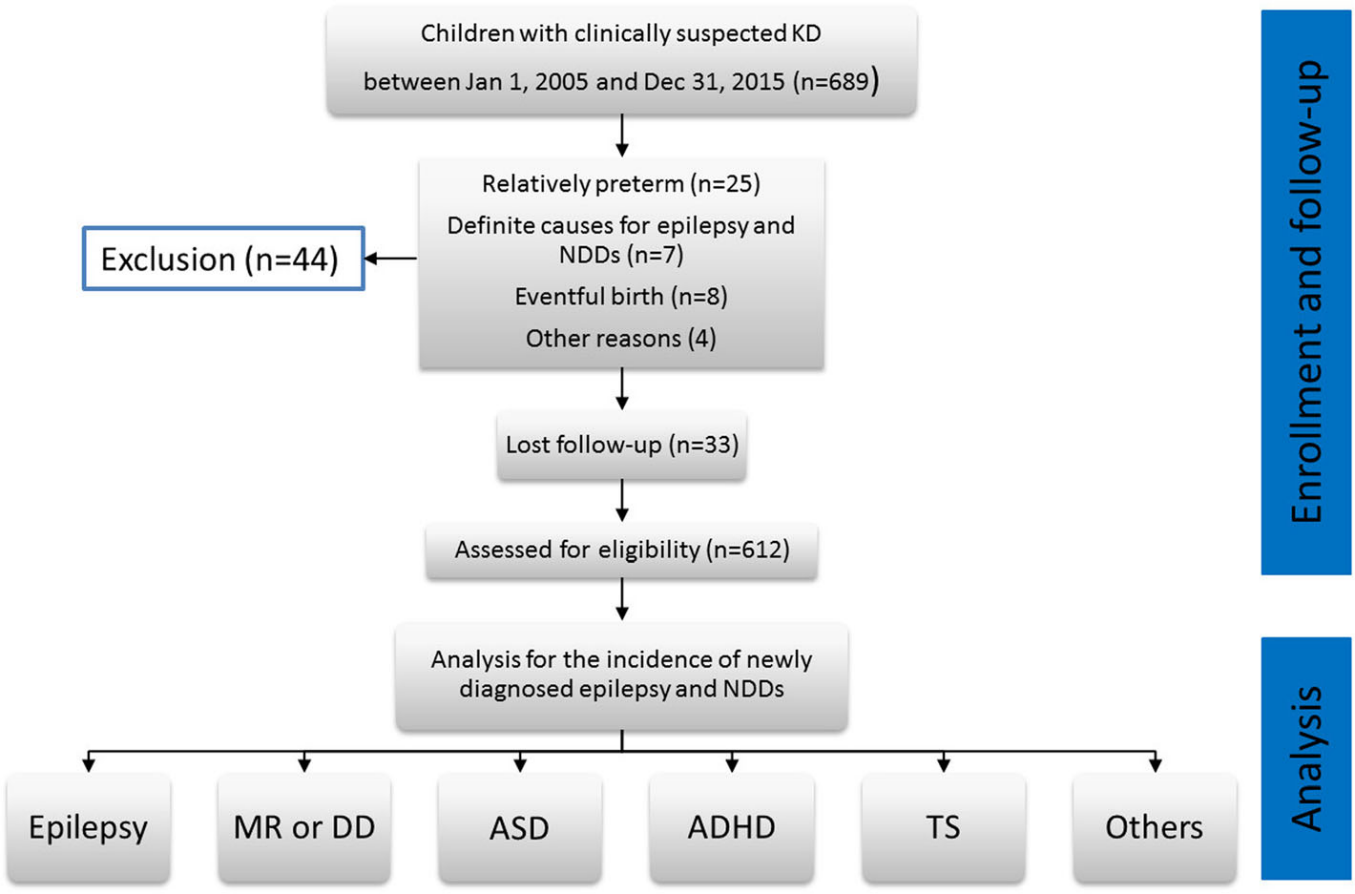
The study flowchart

The last patient was enrolled in December 2015. All patients included in the study were followed up from baseline until the end of follow-up (December 31, 2018), withdrawal from the insurance program, or death. We followed up patients by reviewing their medical records and contacting their families through telephone or e-mail quarterly since the beginning of 2016. Once NDD was suspected, we contacted the children returning to our pediatric neurology clinic for a comprehensive assessment. We compiled statistics and proceeded with the analysis to observe the prevalence of associated NDDs in our study children during 2018. A flowchart of the study is shown in Fig. 1.

Instruments used for assessing children and adolescents with suspected intellectual disability (ID) were Bayley Scales of Infant and Toddler Development, Third Edition (for young children aged < 2 years) and Wechsler Preschool and Primary Scale of Intelligence (for children aged 2 years 6 months to 7 years 7 months). Epilepsy (our outcome of interest) was defined as the occurrence of two unprovoked seizures more than 24 h apart, which was diagnosed by a pediatric neurologist. Patients who met relevant diagnostic criteria in the Fourth and Fifth Editions of Diagnostic and Statistical Manual of Mental Disorders were diagnosed with autism spectrum disorder (ASD) and attention deficit hyperactivity disorder (ADHD); Tourette syndrome (TS) was diagnosed on the basis of the Tourette Syndrome Classification Study Group criteria [11], a guideline for our children’s neurology clinic, and TS was diagnosed by a pediatric psychiatrist or a pediatric neurologist in the inpatient or outpatient setting of China Medical University Children’s Hospital between January 1, 2005, and December 31, 2018.

### Statistical analysis

Although we did not set up a control group for children with KD, we conducted a thorough article review as well as to compare the incidence rates of various NDDs in the Taiwan population and other populations worldwide with those in our study population. Moreover, we randomly select 23,699 children (age 0–18) from National Health Insurance Research Database (NHIRD) during 2015–2016 as a reference to compare the prevalence of various NDDs with our results. The study protocol was approved by the Ethics Review Board of the China Medical University Ethics Committee. All statistical analyses were performed using PASW Statistics version 18.0 software (SPSS Inc., Chicago, IL, USA). In addition, for all executed statistical analyses, we considered two-tailed *p* < 0.05 to indicate statistical significance.

## Results

### Data analysis

Between January 1, 2005, and December 31, 2015, 612 children diagnosed with KD were enrolled in this study. Participants’ demographic factors are presented in Table 1. Participants’ mean age was 1.6 years (standard deviation, 2.4 years). The proportion of boys was higher than that of girls (64.3% vs 35.6%). Positive cardiovascular findings at diagnosis were 48.2%; the proportion of patients who underwent treatment with intravenous immune globulin was 99%.

**Table 1:** Demographic data of children with KD

Demographic data	Children with KD (*n* = 612) (%)
Sex	
Male	394 (64.3)
Female	218 (35.6)
Mean age onset of KD (yrs) (SD)	1.6 ± 2.4
Stratified by age (years)	
0–2	501 (81.8)
2–5	70 (11.4)
5–10	32 (5.2)
> 10	9 (1.4)
Cardiovascular findings at diagnosis	
Positive	295 (48.2)
Negative	317 (51.7)
Treatment with intravenous immune globulin (IVIG; 2 g/kg)	
Yes, within 10 days	538 (87.9)
Yes, over 10 days	67 (10.9)
No	7 (1.1)

### Neurodevelopmental disabilities associated with KD

**Fig. 2:**
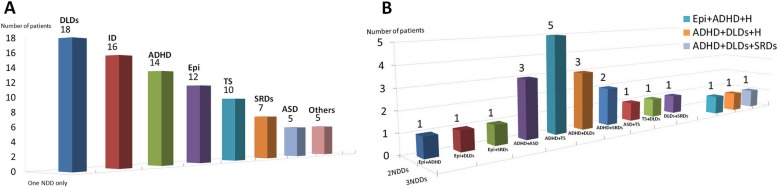
Heterogeneous neurodevelopmental disabilities distributed in single and combined types among children with KD in the study. **a** Children with one NDD only^†^ (**b**) Children with two or more NDDs^‡^. ^**†**‡^Epi, epilepsy; ID, Intellectual disability; ADHD, Attention deficit hyperactivity disorder; ASD, Autism spectrum disorder; DLDs, Developmental language disorders; H, hearing impairment; SRDs, sleep-related disorders; TS, Tourette syndrome.

Associated neurodevelopmental disabilities (*n* = 103/612, 16.8%) in the study were classified as epilepsy, ID, ASD, TS, ADHD, and others (e.g., communication and developmental language disorders). During the follow-up period, few patients developed only one NDD, with the majority being diagnosed with developmental language disorders (*n* = 18), followed by ID (*n*= 16), ADHD (*n*= 14), epilepsy (*n*= 12) and TS (*n*= 10; Fig. 2a**).** Few patients developed two NDDs, with the majority being diagnosed with ADHD + TS (*n* = 5), followed by ADHD + ASD (*n* = 3) and ADHD + developmental language disorders (n = 3). Three individual patients developed more than two NDDs, namely epilepsy + ADHD + hearing impairment, ADHD + developmental language disorders + hearing impairment, and ADHD + developmental language disorders + sleep-associated disorders, respectively (Fig. 2b).

### Comparison of the prevalence of neurodevelopmental disabilities between children with KD and the general population

We compared all and individual NDDs between children with KD and the general pediatric population in Taiwan and worldwide (Table 2) [12–54]. Our data revealed that children with KD had higher prevalence rates of any NDD than did the general pediatric population in Taiwan (16.8% vs 1.3–3% in publications and 5.89% in NHIRD), *p* < 0.05), but this difference has not been significantly observed in other large-scale studies conducted worldwide [12–19]. Similarly, children with KD had a higher prevalence rate of ASD than did the general pediatric population in Taiwan (1.46% vs 0.08–0.29% in publications and 0.41% in NHIRD, *p* < 0.05), but no significant difference was observed between our findings and those reported in other studies conducted worldwide [38–45]. However, children with KD had higher prevalence rates of epilepsy and TS in both Taiwan and worldwide (epilepsy: 2.61% in the KD group vs 0.33% in publication in Taiwan (0.67% in NHIRD) and 0.05–0.8% in worldwide [*p* < 0.05] [24–28]; TS: 2.77% in the KD group vs 0.56% in publication in Taiwan (0.37% in NHIRD) and 0.3–1% in worldwide [*p* < 0.05] [46–50]. Table 3 further compares the possible confounders in children with KD with the development of different neurodevelopmental disabilities. No significant differences were found in terms of whether the children had cardiovascular findings at diagnosis and the timing of IVIG treatment.

**Table 2:** Prevalence of neurodevelopmental conditions for children with KD during the whole year of 2018 versus those in the Taiwan and worldwide

Neurobehavioral conditions (n)	Our children with KD (*n* = 612)	Controls:			
		Taiwan population			Worldwide
	Prevalence	Prevalence (references)	Research type	^†^Prevalence in 2016 based on NHIRD of Taiwan (*n* = 23,699) (%)	Prevalence (references)
Any (102)*	16.8%*	1.3–3% (12)	Meta-analysis	1397 (5.89)	3–18% (13–19)
ID (17)	2.9%	3.25% (20)	Population-based	184 (0.78)	1–3% (21–23)
Epilepsy (16)**	2.61%**	0.33% (24)	Population-based	159 (0.67)	0.05–0.8% (25–28)
ADHD (31)	5.05%	6.3% (29)	Population-based	1110 (4.68)	2–18% (30–37)
ASD (9)*	1.46%*	0.08–0.29% (38, 39)	Meta-analysis	98 (0.41)	0.2–2.5% (40–45)
TS (17)**	2.77%**	0.56% (46)	Large clinic-based	87 (0.37)	0.3–1% (47–50)
DLDs (26)	4.24%	N/A	N/A	1446 (6.1)	5–10% (51–54)

*p < 0.05 in Taiwan population only

**p < 0.05 in both Taiwan and worldwide

^**†**^ID, Intellectual disability; ADHD, Attention deficit hyperactivity disorder; ASD, Autism spectrum disorder; DLDs, Developmental language disorders; TS, Tourette syndrome; NHIRD, National Health Insurance Research Database

**Table 3:** Comparison of prevalence of neurodevelopmental conditions between subgroup of children with KD

Children with KD						
Demographic data	CV^+^ (*n* = 295) (%)	CV^−^(*n* = 317) (%)	*p*-value	IVIG within 10 days (*n* = 538) (%)	IVIG over 10 days (*n* = 67) (%)	*p*-value
ID	7 (2.3)	10 (3.1)	0.76	14 (2.6)	3 (4.4)	0.68
Epilepsy	8 (2.7)	8 (2.5)	0.88	14 (2.6)	2 (2.9)	0.92
ADHD	12 (4.1)	19 (6.0)	0.27	27 (5.0)	4 (5.9)	0.90
ASD	6 (2.0)	3 (0.9)	0.26	8 (1.4)	1 (1.4)	0.90
TS	9 (3.1)	8 (2.5)	0.69	15 (2.7)	2 (2.9)	0.88
DLDs	12 (4.1)	14 (4.4)	0.83	23 (4.2)	3 (4.4)	0.87

^**†**^CV^+^, Positive cardiovascular findings at diagnosis; CV^−^, negative cardiovascular findings at diagnosis; IVIG, intravenous immune globulin

^‡^ID, Intellectual disability; ADHD, Attention deficit hyperactivity disorder; ASD, Autism spectrum disorder; DLDs, Developmental language disorders; TS, Tourette syndrome

## Discussion

The prevalence of NDDs in children ranges from 3 to 18% worldwide; the prevalence varies among different populations and different study designs [13–19]. Although NDDs in children are common and essentially nonfatal, their treatment is expensive and imposes heavy burden not only on patients but also on family and society. In the present study, we found that 16.8% of children with KD developed NDDs at follow-up (3–12 years), which was significantly higher than that in the Taiwan population. Their NDDs were highly variable, including developmental language disorders, followed by ADHD, epilepsy, TS, ID, and sleep-associated disorders. Moreover, we found higher incidences of epilepsy and TS in the study group, which were significant in both Taiwan and worldwide. However, a higher prevalence of ASD was found in the study, but this was not significantly different when compared with worldwide data [38–54].

Whether KD results in long-term neurological problems is still controversial, and few studies have explored their relevance. A retrospective study including 65 patients reported an increase in long-term behavior sequelae in children following KD when compared with hospital- and sibling-matched controls [8]. By contrast, another study showed no difference in the incidence of general physical and psychosocial health in 110 children with KD compared with the general population sample [7]. In Taiwan, a nationwide study indicated that patients with KD may not have an increased risk of ADHD [55]. By contrast, another population-based study in Taiwan showed that ADHD patients had an increased prevalence of various allergic or autoimmune diseases, including asthma, atopic dermatitis, urticaria, ankylosing spondylitis, ulcerative colitis, and autoimmune thyroid disease; however, KD was not included in this study [56]. Kuo et al. [57] investigated the association between KD and autism in Taiwan by using the Taiwan National Health Insurance Research Database and observed no statistical significance between KD and control groups during the 5-year follow-up period. A negative correlation was observed between KD and ID from the analysis of the same database [58].

The correlation between KD and epilepsy and TS is the most noteworthy part of this study. When we conducted a literature review regarding KD and epilepsy or seizures, we found that only few articles have discussed their relevance. Shimakawa et al. described seizure characteristics in the clinical course of KD. A case report mentioned an infant who developed petechia lesions and seizures due to subdural hemorrhage during the acute stage of KD [59]. Acute encephalopathy and seizures were reported to be the initial presentation of KD or preceded the classic symptoms of KD [60]. However, all these aforementioned studies were clinical reports and did not mention the long-term risk of epilepsy. Although we could not determine neuropathogenetic changes occurring in the brain after KD or during the acute stage of KD,our study proposed the correlation between KD and epilepsy through long-term observation. Brain involvement of systemic autoimmune disorders commonly causes seizures as a presenting symptom[61]. Recent studies have shown a trend that many autoimmune disorders, including multiple sclerosis, diabetes mellitus, celiac disease, thyroid disease, systemic lupus erythematosus, antiphospholipid syndrome, rheumatic arthritis, Behçet’s disease and Sjögren syndrome, possibly increase the risk of epilepsy [62]. KD, essentially an autoimmune vasculitis, was indicated in our results to have 8-fold increased risk of epilepsy than the references. This phenomenon echoes recent research that autoimmune disease have been implicated as causative factors of seizures and epilepsy [63, 64]. In addition, when we investigated the association between KD and TS, no associated publication was discovered in PubMed. Thus, the reason for children with KD in this study exhibiting a higher incidence of TS remains unclear. This issue requires further discussion because TS accounts for an important part of NDDs, and KD might pose as a candidate risk factor [65].

Our results partially echoed the hypothesis of previous studies for the negative correlation between KD and other NDDs, such as KD and ADHD, KD and ID, and KD and developmental language disorders. To date, the classification of NDDs is complex and is still considered “in progress” [66]. Hence, the more NDDs are discovered with time and the more we understand the role of autoimmunity in NDDs, the more we are interested in the effect of KD on NDDs [67–69]. Therefore, we assume that KD, possibly a consequence of immunologic response following a systemic, inflammatory illness [70, 71], should also be regarded as a potential risk factor for NDDs. However, future studies may use larger study samples and more in-depth study designs.

This study has some limitations. First, some confounding factors may have affected the results, such as mothers’ medical condition during pregnancy, socioeconomic status, other pharmacological treatment of children with KD, different severities of KD, and other environmental factors. Second, we did not include a control group for our children with KD, the methodology used in other comparative studies was not the same as that used in the present study, and the definition of NDDs used in other comparative studies was not the same as that used in the present study (e.g., for NDDs, other studies used “prevalence, whereas we used “incidence”); in addition, inconsistent observation periods between other studies and our study may have affected final results. Third, because NDDs are a spectrum of conditions with heterogeneity in disease types, geographic incidence, and population incidence, we could not analyze all NDD types and different populations because of the restrictions of study design. Furthermore, additional research focusing on neuroimaging, genetic susceptibility, or proinflammatory cytokines and other environmental exposures may identify potential mechanisms underlying NDDs in children with KD [7].

## Conclusions

The results of this study revealed a higher prevalence rate of NDDs in children with KD in Taiwan. However, these NDDs could be heterogeneous. Among them, children with KD have a higher prevalence of epilepsy and TS remarkably, but no significance difference was observed among ID, ADHD, and developmental language disorders. Children diagnosed with KD should be followed up because they have a higher risk of heterogeneous NDDs. More studies enrolling larger sample sizes and more in-depth study designs containing different cofounders are necessary in the future.

## Data Availability

Datasets are available on request.

## Abbreviations

ADHD: Attention deficit hyperactivity disorder
ASD: Autism spectrum disorders
ID: Intellectual disability
KD: Kawasaki disease
NDDs: Neurodevelopmental disorders
TS: Tourette syndrome
